# Inhibition of Brain GTP Cyclohydrolase I Attenuates 3-Nitropropionic Acid-Induced Striatal Toxicity: Involvement of Mas Receptor/PI3k/Akt/CREB/ BDNF Axis

**DOI:** 10.3389/fphar.2021.740966

**Published:** 2021-12-22

**Authors:** Aya M. Mustafa, Mostafa A. Rabie, Hala F. Zaki, Aya M. Shaheen

**Affiliations:** ^1^ Department of Pharmacology and Toxicology, Faculty of Pharmacy, Egyptian Russian University, Cairo, Egypt; ^2^ Department of Pharmacology and Toxicology, Faculty of Pharmacy, Cairo University, Cairo, Egypt

**Keywords:** 3-nitropropionic acid, mitochondrial dysfunction, mas receptor, PI3K/AKT signaling, DAHP 3

## Abstract

GTP cyclohydrolase I (GTPCH I) is the rate-limiting enzyme for tetrahydrobiopterin (BH4) biosynthesis; the latter is an essential factor for iNOS activation that contributes neuronal loss in Huntington’s disease (HD). The aim of the study was to investigate the neuroprotective effect of 2,4-diamino-6-hydroxypyrimidine (DAHP), GTPCH I enzyme inhibitor, against neuronal loss in 3-nitropropinic acid (3-NP)-induced HD in rats and to reveal the possible involved mechanisms mediated through PI3K/Akt axis and its correlation to Mas receptor (MasR). Rats received 3-NP (10 mg/kg/day; i.p.) with or without administration of DAHP (0.5 g/kg/day; i.p.) or wortmannin (WM), a PI3K inhibitor, (15 μg/kg/day; i.v.) for 14 days. DAHP improved cognitive, memory, and motor abnormalities induced by 3-NP, as confirmed by striatal histopathological specimens and immunohistochemical examination of GFAP. Moreover, DAHP treatment inhibited GTPCH I activity, resulting in decreased BH4 levels and iNOS activation. Also, DAHP upregulated the protein expression of survival protein; p85/p55 (pY458/199)-PI3K and pS473-Akt that, in turn, boosted the activation of striatal neurotrophic factors and receptor, pS133-CREB, BDNF and pY515-TrKB, which positively affect MasR protein expression and improve mitochondrial dysfunction, as indicated by enhancing both SDH and PGC-1α levels. Indeed, DAHP attenuates oxidative stress by increasing SOD activity and Nrf2 expression in addition to reducing neuro-inflammatory status by inhibiting NF-κB p65 and TNF-α expression. Interestingly, all the previous effects were blocked by co-administration of WM with DAHP. In conclusion, DAHP exerts neuroprotective effect against neuronal loss induced by 3-NP administration *via* inhibition of GTPCH I and iNOS activity and activation of MasR/PI3K/Akt/CREB/BDNF/TrKB axis besides its antioxidant and anti-inflammatory effect.

**GRAPHICAL ABSTRACT:**
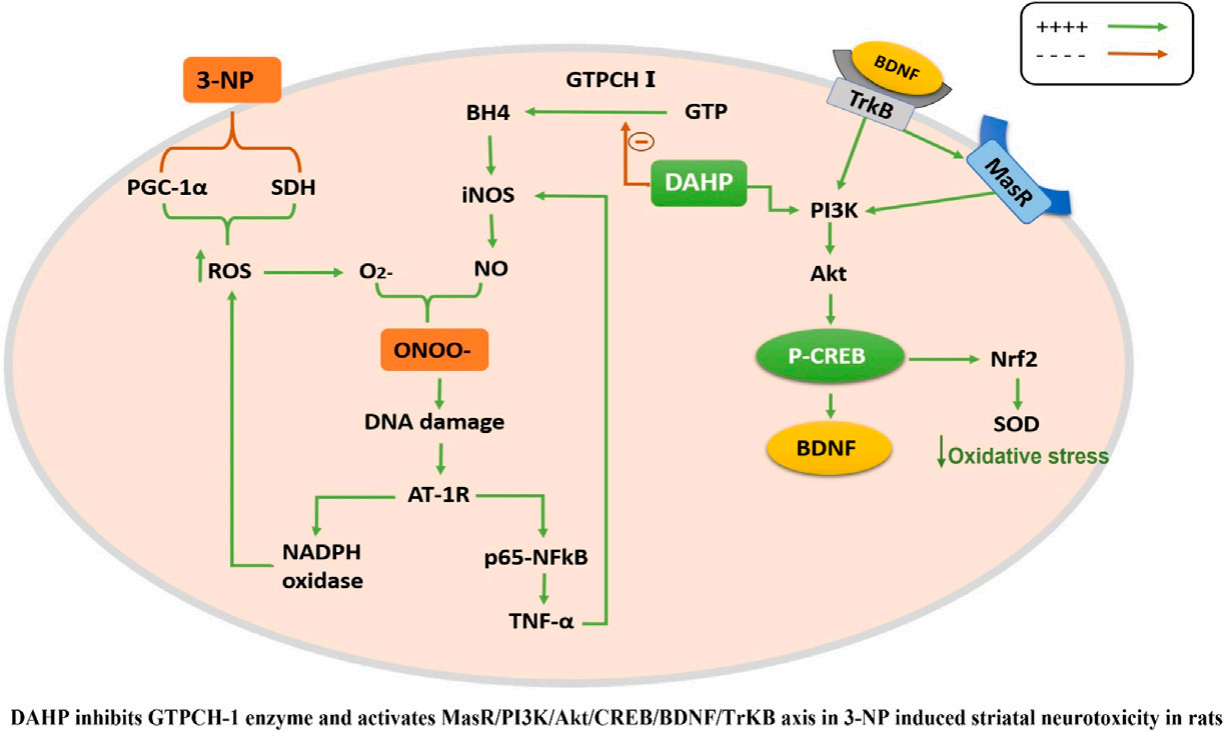


## 1 Introduction

Huntington’s disease (HD) is a progressive neurodegenerative disease characterized by motor and cognitive dysfunctions together with psychiatric manifestations (Shah et al., 1993; Roos, 2010), for which no cure or disease-modifying therapies are available until now (Tabrizi et al., 2019). Of note, inflammation mediated by microglia plays a crucial role in neurodegenerative diseases as Parkinson’s disease (PD) (Gao et al., 2003), Alzheimer’s disease (AD) (Salvati and Beenhakker, 2019), and HD (Sapp et al., 2001). Sustained and excessive activation of microglia along with massive production of proinflammatory cytokines is responsible, in part, for inflammation-induced neurodegeneration (Block et al., 2007; Glass et al., 2010). Nitric oxide (NO) produced by inducible nitric oxide synthase (iNOS) in the microglia is one of the chief proinflammatory factors that induce neuronal death (Liu et al., 2012). Thus, iNOS inhibition attenuated microglia-mediated neuronal death, revealing the pivotal role of NO in microglia-mediated neurotoxicity (Mander and Brown, 2005). Tetrahydrobiopterin (BH4), an essential cofactor for NOS activity and phenylalanine hydroxylase, tryptophan hydroxylase, and tyrosine hydroxylases (Thony et al., 2000). Tyrosine hydroxylase, the rate-limiting enzyme for dopamine biosynthesis, uses tetrahydrobiopterin and molecular oxygen to convert tyrosine to L-DOPA (Thony et al., 2000). GTP cyclohydrolase I (GTPCH I) is the rate- limiting enzyme and the first step in BH4 biosynthesis (Alp and Channon, 2004). Indeed, GTPCH I activation by various cytokines, such as tumor necrosis factor α (TNFα) and interferon γ (IFN-γ) is accompanied by increased BH4 level and NOS activity (Huang et al., 2005). In the present study, 3-nitropropionic acid (3-NP), irreversible inhibitor of mitochondrial succinate dehydrogenase, is used to mimic the pathological and motor abnormalities of HD (Brouillet et al., 2005; Kumar et al., 2011; Gao et al., 2015). 3-NP induces an oxidative stress status and impairs antioxidant defense mechanisms in the brain (Pérez-De La Cruz et al., 2009; Zafir et al., 2009) together with the production of proinflammatory cytokines, such as TNF-α, interleukin-6, and interleukin-1β (Jamwal and Kumar, 2016). The overproduction of reactive oxygen species and neuroinflammatory status results in marked elevation of iNOS activity and peroxynitrite (ONOO-) level, ultimately causing neuronal death (Pedraza-Chaverrí et al., 2009).

Noteworthy, the PI3K/Akt axis exerts a neuroprotective effect in neurodegenerative diseases, such as ischemic stroke, PD, and AD *via* enhancement of cAMP-responsive element-binding protein (CREB) expression with subsequent downstream protein, brain-derived neurotrophic factor (BDNF) leading to cellular proliferation and inhibition of apoptotic and inflammatory biomarkers that eventually ends up with improved cell survival (Jiang et al., 2013; Heras-Sandoval et al., 2014; Ribeiro et al., 2014; Zuo et al., 2016). Moreover, Sayed et al. (2020) reported the crucial function for PI3K and Akt proteins as cellular components in hampering HD (Sayed et al., 2020). It is previously reported that DAHP, GTPCH I inhibitor, demonstrated a neuroprotective effect in focal cerebral ischemia through activation of phosphoinositide-3-kinase/protein kinase B (PI3K/Akt) pathway (Li et al., 2015).

Based on the above data, the current study investigated the neuroprotective effect of 2, 4-diamino-6-hydroxypyrimidine (DAHP), brain GTPCH-1 inhibitor, against neuronal loss in 3-NP induced HD *via* iNOS inhibition. Moreover, the aim was extended to study the protective role of PI3K/Akt axis and its consequence on Mas receptor (MasR) activation against 3-NP induced neurotoxicity using wortmannin (WM) as a direct PI3K pathway inhibitor.

## 2 Materials and Methods

### 2.1 Ethics Statement

The investigation complies with the Guide for the Care and Use of Laboratory Animals published by the US National Institutes of Health (NIH publication No. 8023, revised 1978) adopted by the Ethics Research Committee of Faculty of Pharmacy, Cairo University (Cairo, Egypt; PT (2573). All efforts were done to minimize animal suffering during the experiment.

### 2.2 Animals

Male Wistar rats, weighing 180–200 g, were obtained from the animal facility of Faculty of Pharmacy, Cairo University (Cairo, Egypt). Before starting the experiment, the animals were allowed to acclimatize to laboratory conditions for 1 week. The animals were housed under controlled environmental conditions at constant temperature (23 ± 2°C), humidity (60 ± 10%), and a 12-/12-h light/dark cycle. The rats were allowed free access to standard chow diet and water *ad libitum*, and all behavioral tests were carried out in a sound isolated laboratory.

### 2.3 Experimental Design

**SCHEME 1:**
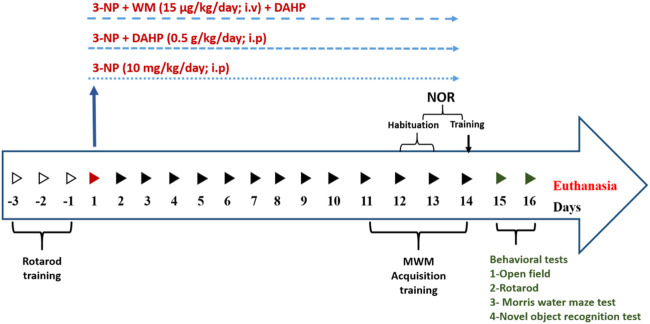
Time schedule for DAHP and wortmannin administration and behavioral assessment in 3-Nitropropionic acid rat model.

The rats were randomly divided into five groups, (*n* = 14/group): Group I received dimethyl sulfoxide (DMSO) (0.2 ml/kg/day; i.p; Thermo Fisher, United States) and served as normal control group. Group II received DAHP (0.5 g/kg/day; i.p; Sigma-Aldrich, MO, United States) (Li et al., 2015) and served as normal drug group. Group III received 3-NP (10 mg/kg/day; i.p; Sigma-Aldrich, MO, United States) (Kumar et al., 2010). Group IV was treated with DAHP (0.5 g/kg/day; i.p; (Li et al., 2015) 1 h after 3-NP injection. Group V was treated with WM (15 μg/kg/day; i.v.; Sigma-Aldrich, MO, United States) after 3-NP injection and 15 min prior to DAHP administration (Yue et al., 2005). All treatments were conducted for 14 days, where 3-NP was dissolved in saline, and the pH was adjusted to 7.4 with NaOH. Meanwhile, DAHP and WM were dissolved in DMSO (Merck, Germany), and then WM was freshly diluted with saline. The animals were subjected to behavioral tests then further classified randomly into three subsets: first subset (*n* = 5) was used to assess parameters by Western blot technique, the second subset (*n* = 6) was used to measure parameters by ELISA technique, and the third subset (*n* = 3) was used for striatal histopathological examination and immunohistochemical assessment of glial fibrillary acidic protein (GFAP). The following Scheme 1 summarizes the timeline for behavioral tests and treatments.

### 2.4 Behavioral Tests

Twenty-four hours after the last injection of 3-NP, DAHP, and/or WM, the rats were screened for motor performance using the open field and rotarod test. Additionally, memory was assessed using Morris water maze test and novel object recognition test. The tests were conducted on two consecutive days during the light cycle, Day 1) Open field and Rotarod test were performed and on the second day, Morris Water Maze and Novel object recognition tests were performed with 2-h respite period between the tests (Ramachandran and Thangarajan, 2016; Sayed et al., 2020).

#### 2.4.1 Open Field Test

Open field test was carried out to assess spontaneous locomotor activity. The apparatus was a square box (80 × 80 × 40 cm) made of wood with red walls and black polished floor divided by white lines into 16 equal squares. The rats were individually placed at the center of the apparatus and allowed to explore the field for 5 min. An overhead camera was used to monitor the animals, and record ambulation frequency (the number of squares crossed by each rat) and rearing frequency (the number of rearings on the hind limbs). After each animal was tested, the floor was cleaned (Ramachandran and Thangarajan, 2018).

#### 2.4.2 Rotarod Test

Motor coordination and grip strength were evaluated using rotarod apparatus (120-cm long, 3 cm in diameter, subdivided into four compartments by disks 24 cm in diameter and rotating at a constant speed of 20 rpm). For 3 days before experimental procedures, the animals were subjected to training sessions where the animal that continued on the rod for 5 min was chosen to carry out the experiment. After completion of OFT, the test was performed and fall off latency was recorded (Avila et al., 2010).

#### 2.4.3 Morris Water Maze

The rats were screened for memory retention and spatial learning using the Morris water maze. The animals were trained to swim to a platform in a circular pool (150 cm in diameter and 60 cm in height with non-reflective interior surfaces) divided into four quadrants and filled with water up to 35-cm level and at a constant temperature of 25 ± 2°C. A movable circular platform (9 cm in diameter) was placed in the center of specific quadrant of the pool 1 cm below the water surface for acquisition test. A non-toxic soluble black paint was used to make the water opaque. Initially, the rats were subjected to three training sessions per day, each 120 s, for 4 days, in which the animals were left freely to find the platform from different starting positions. If the rat did not find the platform it was guided to it, and left on it for 30 s. Average time taken by the rat to reach the platform was recorded as acquisition latency. On the fifth day, a probe test was performed where the platform was removed and the animal was released facing the wall of the pool at quadrant opposite to the target quadrant, and was allowed to explore the pool for 1 min. The time spent by the animal swimming in the target quadrant was recorded using overhead camera (Suganya and Sumathi, 2017).

#### 2.4.4 Novel Object Recognition Test

Novel object recognition test was performed to evaluate cognition and particularly recognition memory. The test was carried out in a black open field box measuring 50 × 25 × 50 cm. During habituation, the rats were allowed to explore the test box with no objects present for 10 min per day for two consecutive days. In the training sessions, each rat was placed in the test box with two identical objects placed in two corners (approximately 30-cm apart from each other). On the test day, the animals were introduced back in the test box, in which one of the familiar objects was replaced with a novel object. The time spent exploring each object was recorded for 3 min using overhead camera during training and test sessions (Karasawa et al., 2008; Chen et al., 2019). Discrimination index, the difference in time spent exploring familiar and novel objects over the total time spent exploring both objects was calculated, and the time spent exploring familiar and novel objects as well (Arnt et al., 2010; Antunes and Biala, 2012).

### 2.5 Striatal Processing

At the end of behavioral tests, the rats were weighed and euthanized where the whole brains were quickly excised, washed with ice-cold saline, and dissected. Striata from each brain were immediately isolated and flash frozen in liquid nitrogen, then stored at −80°C.

#### 2.5.1 Measurement of Tetrahydrobiopterin Levels and GTP Cyclohydrolase I Activity

BH4 levels and GTPCH I activity were assessed by high-performance liquid chromatography (HPLC) analysis using fluorescence detection as previously described (d’Uscio et al., 2003). Total biopterins including BH4 plus dihydropterin (BH2) plus oxidized biopterins were determined by acid oxidation, whereas BH2 and oxidized biopterins were determined by alkali oxidation. BH4 content was calculated from the difference between total biopterins to BH2 plus oxidized biopterins. GTPCH I activity was assessed using HPLC method with measurement of neopterin, after oxidation and phosphatase treatment of dihydroneopterin triphosphate.

#### 2.5.2 Western Blot Analysis of Mas receptor, p85/p55 (pY458/199)-PI3K, pS473-Akt, pS133-cAMP Responsive Element-Binding Protein, Brain-Derived Neurotrophic Factor, pY515-TrkB, and Nuclear Factor Erythroid-2-Related Factor-2

The striata in the first subset were homogenized in radio immunoprecipitation assay (RIPA) buffer (150 mM NaCl, 1% Triton X-100, 0.5% sodium deoxycholate, 50 mM Tris HCl pH 8, and 0.1% SDS) supplied with freshly made protease–phosphatase inhibitors cocktail to maintain protein integrity. Bradford Protein Assay Kit (Bio BASIC Inc., ON, Canada) was used for quantitative striatal protein analysis. A 10-µg protein concentration of each sample was boiled with Laemmli buffer for 5 min and separated by SDS-PAGE and transferred to PVDF membrane that was blocked with 5% bovine serum albumin (BSA). Protein expression was evaluated by incubating membrane with primary antibodies (Thermo Fisher Scientific, MA, United States) against Mas receptor (0.25 µg/ml; cat#: PA5-43669), p85/p55 (pY458/199)-PI3K (1:1,000; cat#: PA5–17387), pS473-Akt (1:250; cat#: 700392), pS133-CREB (1:250; cat#: PA1-851B), BDNF (1:1,000; cat#: OSB00017W), pY515-TrkB (1:2,500; cat#: PA5–38076), nuclear factor erythroid-2-related factor-2 (Nrf2) (1:1,000; cat#: PA5–67520), and β-actin (1:1,000; cat#: PA5-16914) polyclonal antibody overnight at 4°C. Afterward, membranes were probed with horseradish peroxidase-conjugated goat anti-rabbit immunoglobulin (Dianova, Hamburg, Germany) for 2 h at room temperature. The amount of target proteins was quantified by densitometric analysis using Image analysis software on the ChemiDoc™ MP Imaging System (version 3) (Bio-Rad, CA, United States). The percentage of acrylamide used for all studied protein was 10% except for Nrf2 and TrKB, with a percentage of 8%. Results were normalized for β-actin protein expression and expressed as arbitrary units (AU).

#### 2.5.3 ELISA Assay of Striatal Proliferator-Activated Receptor Gamma Coactivator 1-Alpha, Tumor Necrosis Factor-Alpha , Inducible Nitric Oxide Synthase , Nuclear Factor-κB, Succinate Dehydrogenase , and Superoxide Dismutase

Striata were rinsed and homogenized in PBS for quantitative determination of proliferator-activated receptor gamma coactivator 1-alpha (PGC-1α) (cat#: CSB-EL018425RA) and tumor necrosis factor-alpha (TNF-α) (cat#: CSB-E11987r) using CUSABIO ELISA kits (Wuhan, PRC). The MyBioSource ELISA kits (CA, United States) were used to determine iNOS (cat#: MBS263618), nuclear factor- κB p65 (NF-κB p65) (cat#: MBS015549), SDH (cat#: MBS3807968) and superoxide dismutase (SOD) (cat#: MBS036924). The procedures were performed according to the instructions of the manufacturer and the results were presented as pg/mg tissue protein for PGC-1α, TNF-α and NF-κB p65, ng/mg tissue protein for iNOS and SDH and U/mg tissue protein for SOD.

### 2.6 Histopathological Examination

Tissue samples were fixed in 10% neutral buffered formalin for 72 h with a change of formalin solution every day. The samples were washed, dehydrated, and processed in serial grades of ethanol, cleared in Xylene, synthetic wax infiltration, and embedded into Paraplast tissue embedding media. The 5-μm-thick sagittal brain sections were cut by rotatory microtome, stained with hematoxylin and eosin (H&E) and examined under light microscope for demonstration of striatal regions in different samples (Sidhu et al., 2018). All micrographs and data were obtained by using full HD microscopic camera operated by Leica application module for histological analysis (Leica Microsystems GmbH, Wetzlar, Germany) (Sayed et al., 2020).

### 2.7 Immunohistochemical Detection of Glial Fibrillary Acidic Protein

Deparaffinized 5-μm-thick tissue sections were cut and prepared for evaluation of astroglial alteration. Striatal sections were treated with 3% hydrogen peroxide for 20 min, washed with PBS, then incubated with mouse monoclonal glial fibrillary acidic protein (GFAP) antibody (Thermo Fisher Scientific Inc., United States) for 30 min. The sections were washed with PBS followed by incubation for 20 min with secondary antibody (Dako, Carpenteria, CA, United States), and then with horseradish peroxidase using the HRP Envision kit (Dako, Carpenteria, CA, United States). The reaction was visualized with 3,3′-diaminobenzidine tetrahydrochloride (DAB Substrate Kit, Vector Laboratories Inc., Burlingame, CA, United States) for 10 min following another wash with PBS. Finally, the sections were counterstained with hematoxylin, dehydrated, and cleared in xylene then cover-slipped for microscopic analysis. Six randomly selected fields from striatum region were analyzed for determination of GFAP immunoreactive percentage areas in individual sections using full HD microscopic camera operated by Leica application module for histological analysis (Sayed et al., 2020).

### 2.8 Statistical Analysis

All data obtained were expressed as mean ± SD. Results were analyzed using one-way analysis of variance test (one-way ANOVA) followed by Tukey’s multiple comparison test for all parameters, except ambulation frequency and rearing frequency which were analyzed using Kruskal–Wallis test followed by Dunn’s multiple comparison test. Differences in familiar and novel object exploration time for each group were tested for significance by two-way ANOVA using objects and drug treatments as set variables. Statistical analysis was performed using GraphPad Prism software (version 5). For all statistical tests, statistical significance was set at *p* < 0.05.

## 3 Results

For all measured parameters, no significant differences were recorded between control group and DAHP group; hence, comparisons were made relative to the control group only.

### 3.1 Effect of 2,4-Diamino-6-Hydroxypyrimidine on Behavioral and Motor Alteration as Well as Body Weight in 3-Nitropropinic Acid Rat Model

**FIGURE 1:**
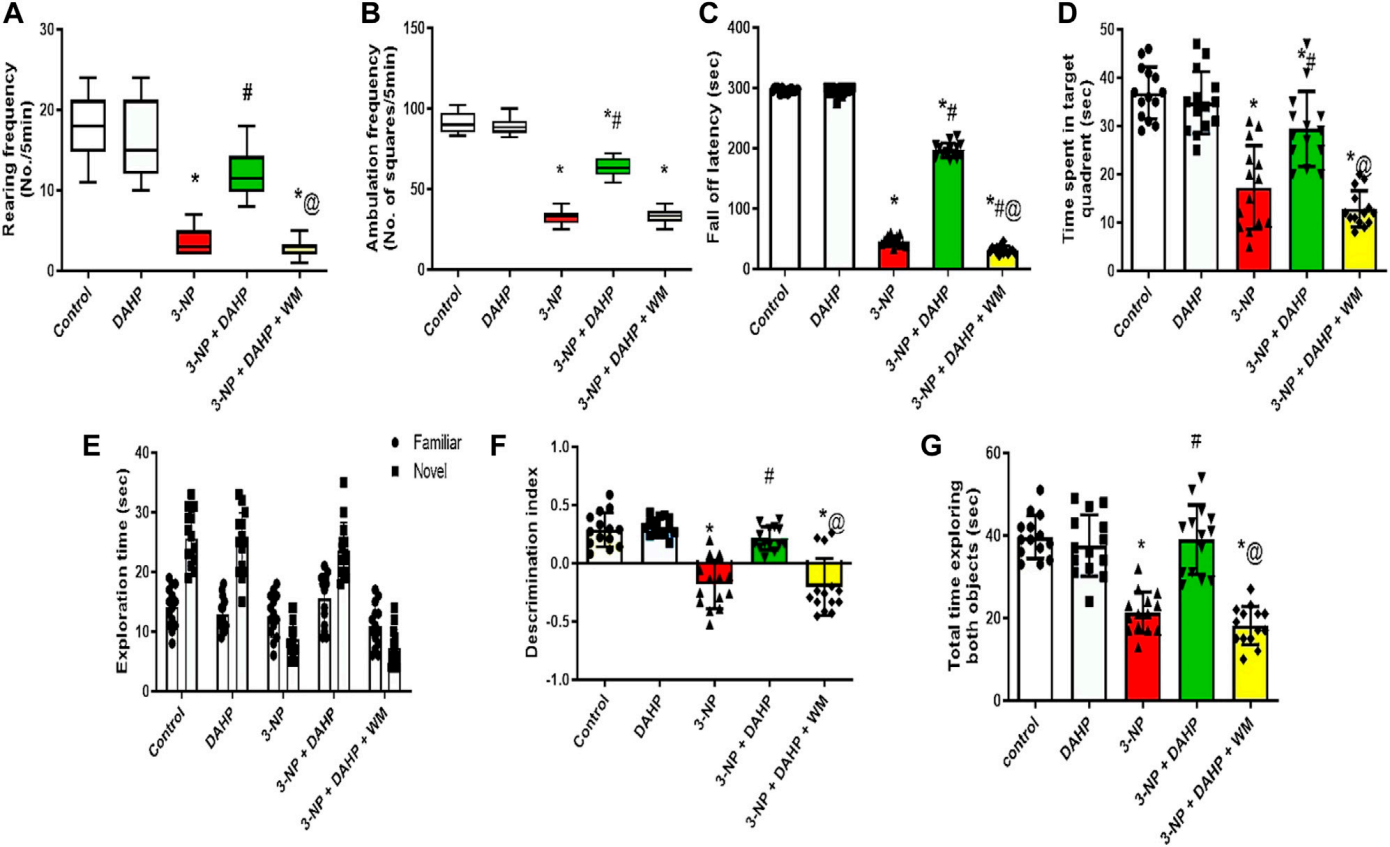
Effect of DAHP on 3-NP-induced changes in **(A)** rearing frequency and **(B)** ambulation frequency in open field test and **(C)** fall off latency in rotarod test, as well as **(D)** time spent in target quadrant in Morris water maze test, **(E)** the exploration time of familiar and novel objects, **(F)** discrimination index and **(G)** Total time exploring both object in novel object recognition test. Rearing and ambulation frequency are presented as boxplots with median, 25th, and 75th percentile values using Kruskel–Wallis test followed by Dunn’s as a *post-hoc* test. Parametric data are presented as mean ± SD of 14 rats per group, using one-way ANOVA followed by Tukey’s *post-hoc* test. Differences in familiar and novel object exploration time for each group were tested using two-way ANOVA; F (4.65) = 3,667, F (4.65) = 36.3, F (4.65) = 32.7, F (4.65) = 44.7; *p* < 0.05. * vs. control, # vs. 3-NP, @ vs. 3-NP + DAHP. 3-NP, 3-nitropropionic acid; DAHP, 2,4-diamino-6-hydroxypyrimidine; WM, wortmannin.

Huntington’s disease (HD) displayed striatal dysfunction was associated with motor and cognitive impairment. In 3-NP rats, marked reduction in rearing frequency and ambulation frequency to 83.33 and 69.44%, respectively, showed in open field test, in addition to decrease in fall of latency to 84.39% in rotarod test. DAHP treatment reversed 3-NP effect that was demonstrated as an increase in rearing frequency and ambulation frequency by 3.8- and 2.5-fold, respectively, in open field test as well as increase in fall of latency by 4.3-fold in rotarod test. In the Morris water maze test, time spent in the target quadrant was reduced to 53.09% in 3-NP group. Furthermore, in novel object recognition test, 3-NP treated rats spent significantly less time exploring the novel object than familiar object (31.1%) and less time exploring the novel object in comparison with control group (65.97%) and showed significant decrease in discrimination index and the total time spent exploring both objects (46.12%) that was a clear indication on cognitive deficit. On the contrary, DAHP treated rats showed significant increase in time spent at the target quadrant by 1.7-fold in the Morris water maze test. This was accompanied by increase in time spent in exploring the novel object than the familiar one by 1.5-fold and increase in time spent in exploring the novel object compared with the 3-NP treated group by 2.7-fold and significant increase in discrimination index and the total time spent exploring both objects by 1.8-fold. Meanwhile, WM pretreatment abolished locomotor and behavioral modulation induced by DAHP in all previous tests (Figure 1).

Regarding body weight, 3-NP reduced the body weight to 15.47% compared with the control group. On the other hand, treatment with DAHP alleviated 3-NP induced body weight loss by 1.16-fold compared with 3-NP group. However, pre-treatment with WM reversed the effect of DAHP in 3-NP rats (Table 1).

**TABLE 1:** Effect of DAHP on 3-NP induced change in body weight.

Group	Final body weight (g)
Control	206.8 ± 7.100
DAHP	205.1 ± 6.184
3-NP	174.8 ± 4.522^a^
3-NP + DAHP	202.7 ± 5.427^a^ ^,^ ^b^
3-NP + DAHP + WM	172.9 ± 4.032^a^ ^,^ ^c^

Note. Data are presented as means ± SD, of 14 rats per group. Statistical analysis was performed using one-way ANOVA, followed by Tukey’s *post hoc* test; F (4.65) = 132; *p* < 0.05. 3-NP, 3-nitropropionic acid; DAHP, 2, 4-diamino-6-hydroxypyrimidine; WM, wortmannin.

a *p* < 0.05 vs. control.

b *p* < 0.05 vs. 3-NP.

c *p* < 0.05 vs. 3-NP + DAHP.

### 3.2 Effect of 2,4-Diamino-6-Hydroxypyrimidine on Striatal GTP Cyclohydrolase I Activity, Tetrahydrobiopterin, and Inducible Nitric Oxide Synthase

**FIGURE 2:**
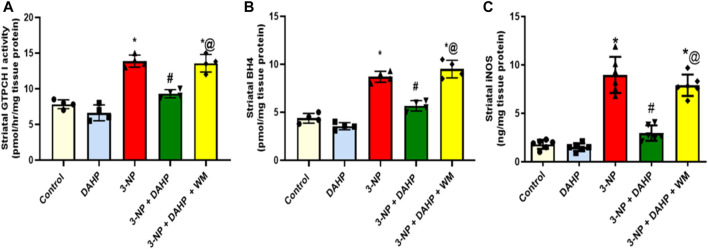
Effect of DAHP on 3-NP induced alteration in striatal **(A)** GTPCH I activity, **(B)** BH4 and **(C)** iNOS level. Data are presented as mean ± SD of four to six rats per group, using one-way ANOVA followed by Tukey’s *post-hoc* test; F (4.15) = 52.5, F (4.15) = 75, F (4.25) = 65.6; *p* < 0.05. * vs. control, # vs. 3-NP, @ vs. 3-NP + DAHP. 3-NP, 3-nitropropionic acid; DAHP, 2, 4-diamino-6-hydroxypyrimidine; WM, wortmannin; GTPCH I, GTP cyclohydrolase I; BH4, tetrahydrobiopterin; iNOS, inducible nitric oxide synthase.

The current study investigated for the first time the possible role of GTPCH I enzyme, the rate-limiting step in BH4 biosynthesis, on iNOS regulation in HD rat model. The 3-NP induced robust increase in GTPCH I activity, BH4 content, and iNOS content to 1.78-, 1.99-, and 4.97-fold, respectively, as compared with control group. On the other hand, treatment with DAHP, GTPCH I inhibitor, showed marked inhibition in GTPCH I, BH4, and iNOS by 33.60%, 34.82%, and 66.99%, respectively, compared with 3-NP group. These effects were reversed by the coadministration of WM with DAHP (Figure 2).

### 3.3 Effect of 2,4-Diamino-6-Hydroxypyrimidine on Striatal Succinate Dehydrogenase, Proliferator-Activated Receptor Gamma Coactivator 1-Alpha, Superoxide Dismutase, and Nuclear Factor Erythroid-2-Related Factor-2 in 3-Nitropropinic Acid Rat Model

**FIGURE 3:**
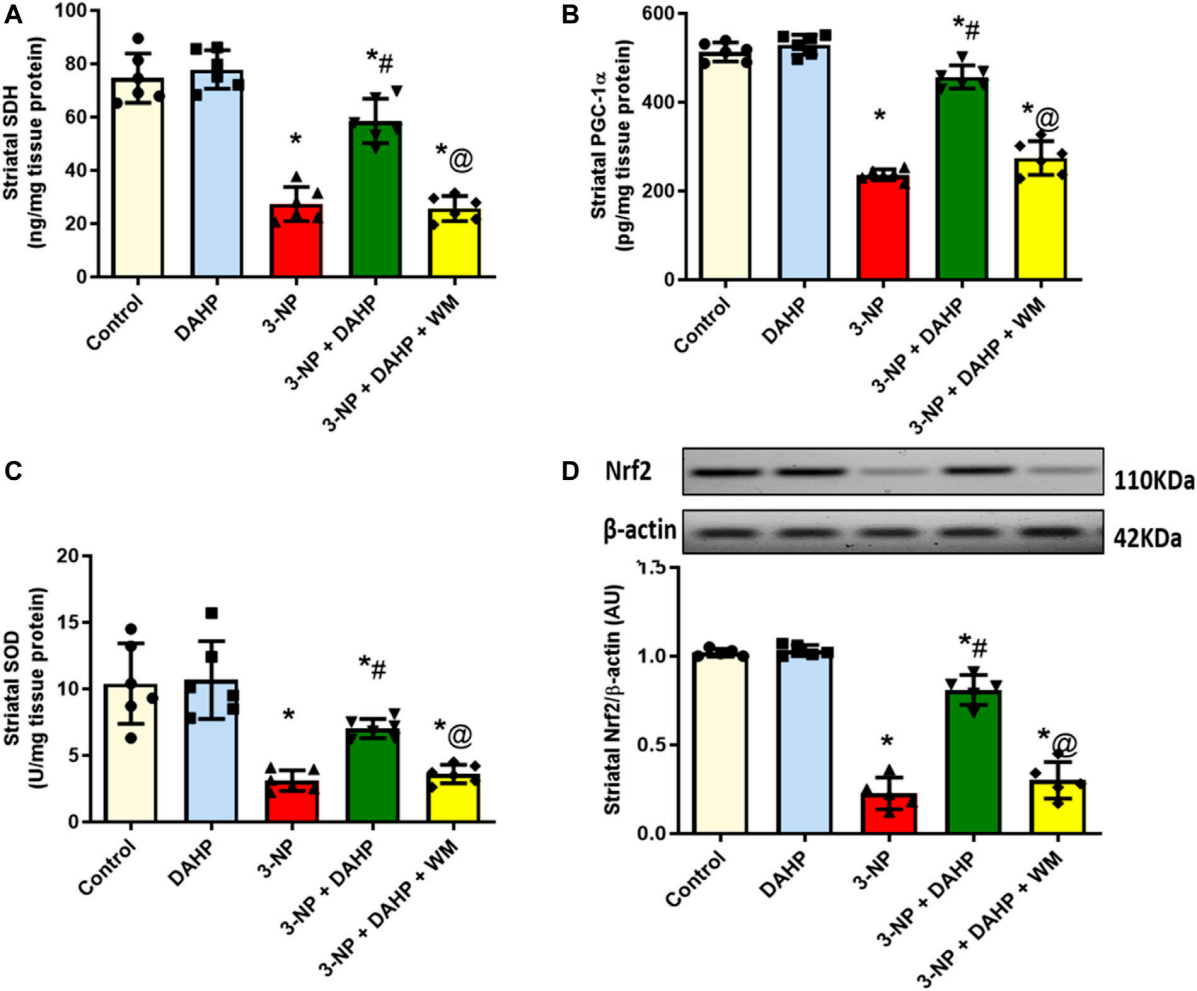
Effect of DAHP on 3-NP induced alteration in striatal **(A)** SDH level, **(B)** PGC-1α content, **(C)** SOD activity and **(D)** Nrf2 protein expression. Data are presented as mean ± SD of five to six rats per group, using one-way ANOVA followed by Tukey’s post hoc test; F (4.25) = 69.6, F (4.25) = 175.9, F (4.25) = 20.1, F (4.20) = 138.8; *p* < 0.05. * vs. control, # vs. 3-NP, @ vs. 3-NP + DAHP. 3-NP, 3-nitropropionic acid; DAHP, 2,4-diamino-6-hydroxypyrimidine; WM, wortmannin; SDH, succinate dehydrogenase; PGC-1α, proliferator-activated receptor gamma coactivator 1-alpha; SOD, superoxide dismutase; Nrf2, nuclear factor erythroid-2-related factor-2.

The 3-NP intoxication showed severe mitochondrial dysfunction together with oxidative stress status demonstrated as significant reduction in striatal SDH level and PGC-1α protein expression as well as SOD and Nrf2 to reach 63.33, 54, 70.22, and 77.82%, respectively, compared with the control group. DHAP improved mitochondrial dysfunction and attenuated oxidative stress; displayed as significant increase in SDH level, PGC-1α protein expression, as well as SOD and Nrf2 by 2.1-, 1.9-, 2.3-, and 3.6-fold compared with 3-NP group. However, WM pre-treatment revoked DAHP-induced modification (Figure 3).

### 3.4 Effect of 2,4-Diamino-6-Hydroxypyrimidine on Striatal Neuroinflammatory Markers in 3-Nitropropinic Acid Rat Model

**FIGURE 4:**
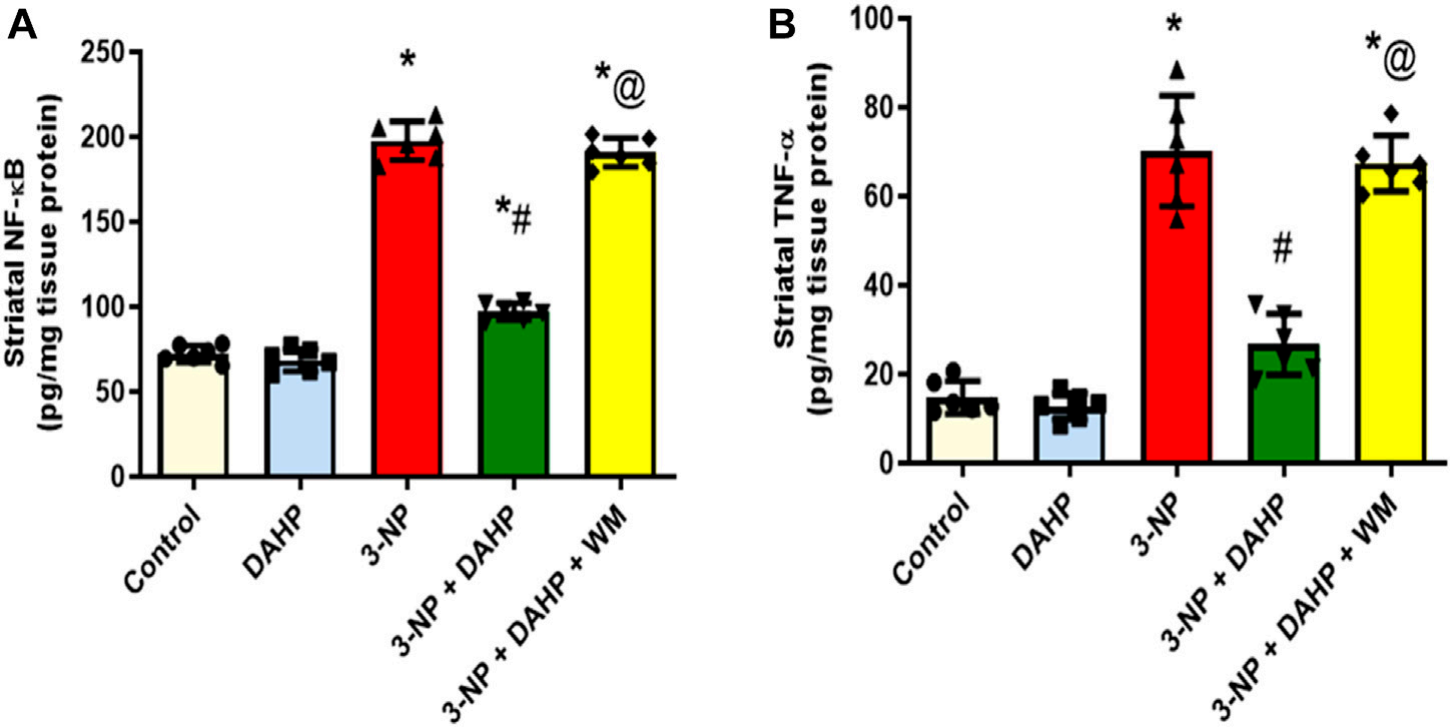
Effect of DAHP on 3-NP induced alteration in striatal **(A)** p65 NF-κB and **(B)** TNF-α content. Data are presented as mean ± SD of six rats per group, using one-way ANOVA followed by Tukey’s *post-hoc* test; F (4.25) = 418, F (4.25) = 91.2; *p* < 0.05. * vs. control, # vs. 3-NP, @ vs. 3-NP + DAHP. 3-NP, 3-nitropropionic acid; DAHP, 2,4-diamino-6-hydroxypyrimidine; WM, wortmannin; p65 NF-kB, p65 nuclear factor-kB; TNF-α, tumor necrosis factor-alpha.

The 3-NP-induced neuroinflammatory status is demonstrated as a significant elevation of p65 nuclear factor-κB (p65 NF-κB) protein expression and tumor necrosis factor-alpha (TNF-α) level by 1.73- and 3.75-fold, respectively, compared with the normal rats. Furthermore, this elevation was abolished by DAHP administration to 49.13 and 38.05% relative to p65 NF-κB and TNF-α, respectively. On the other hand, WM pre-treatment largely obliterated DAHP anti-inflammatory effect and caused 1.96- and 2.52-fold rise in p65 NF-κB and TNF-α, respectively (Figure 4).

### 3.5 Effect of 2,4-Diamino-6-Hydroxypyrimidine on Striatal Contents of Mas Receptor, Phosphoinositide-3-Kinase, Phosphorylated-Serine-Threonine Kinase, Phosphorylated cAMP-Responsive Element-Binding Protein, Brain-Derived Neurotrophic Factor, and Phosphorylated Tyrosine Kinase B in 3-Nitropropinic Acid Rat Model

**FIGURE 5:**
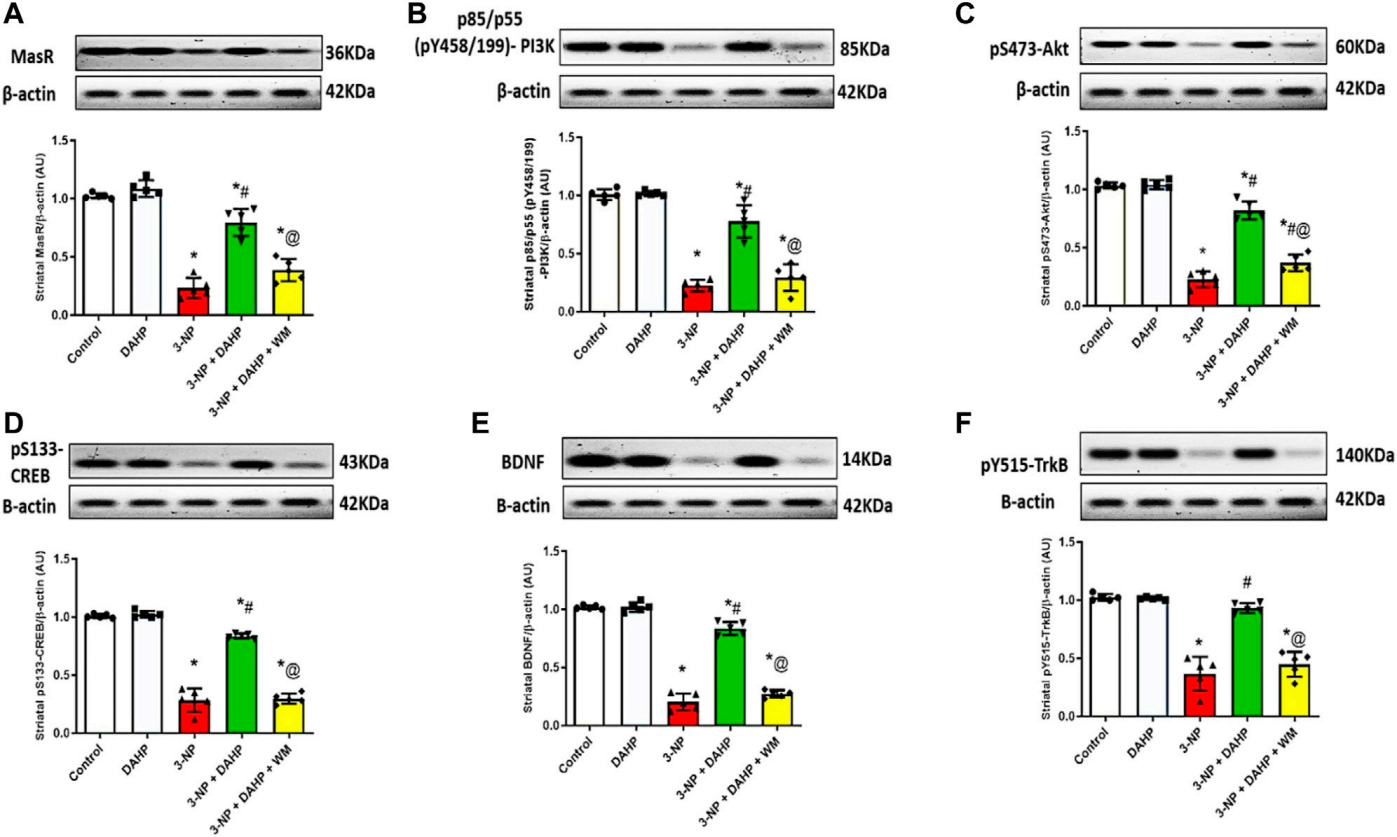
Effect of DAHP on 3-NP induced alteration in striatal **(A)** MasR, **(B)** p-PI3K, **(C)** p-Akt, **(D)** p-CREB, **(E)** BDNF and **(F)** p-TrkB protein expression. Data are presented as mean± SD of five rats per group, using one-way ANOVA followed by Tukey’s *post hoc* test; F (4.20) = 100.4, F (4.20) = 96.7, F (4.20) = 200.5, F (4.20) = 254.4, F (4.20) = 359.7, F (4.20) = 73.6; *p* < 0.05. * vs control, # vs 3-NP, @ vs 3-NP + DAHP. 3-NP, 3-nitropropionic acid; DAHP, 2, 4-diamino-6-hydroxypyrimidine; WM, wortmannin; MasR, Mas receptor; p-P13K, phosphorylated phosphoinositide-3-kinase; p-Akt, phosphorylated protein kinase B; p-CREBm phosphorylated cAMP responsive element-binding protein; BDNF, brain-derived neurotrophic factor; p-TrkB, phosphorylated tyrosine kinase B.

To assess the possible DAHP-induced neuroprotective effect on striatal MasR signaling, it was necessary to assess MasR, and its down streaming signal transduction. In Figure 5, 3-NP caused a marked reduction in MasR protein expression together with the phosphorylated forms of PI3K, Akt, CREB, BDNF, and TrKB to 77.18, 77.82, 78.04, 71.88, 80.04, and 64.26%, respectively, compared with normal values. Oppositely, DAHP treatment elevated MasR, p-PI3K, p-Akt, p-CREB, BDNF, and p-TrKB receptor protein expression by 3.41-, 3.47-, 3.62-, 2.95-, 4.11-, and 2.5- fold, respectively, compared with the insult. This elevation was blocked by coadministration of WM with DAHP.

### 3.6 Effect of 2,4-Diamino-6-Hydroxypyrimidine on 3-Nitropropinic Acid Induced Striatal Histopathological Alterations

**FIGURE 6:**
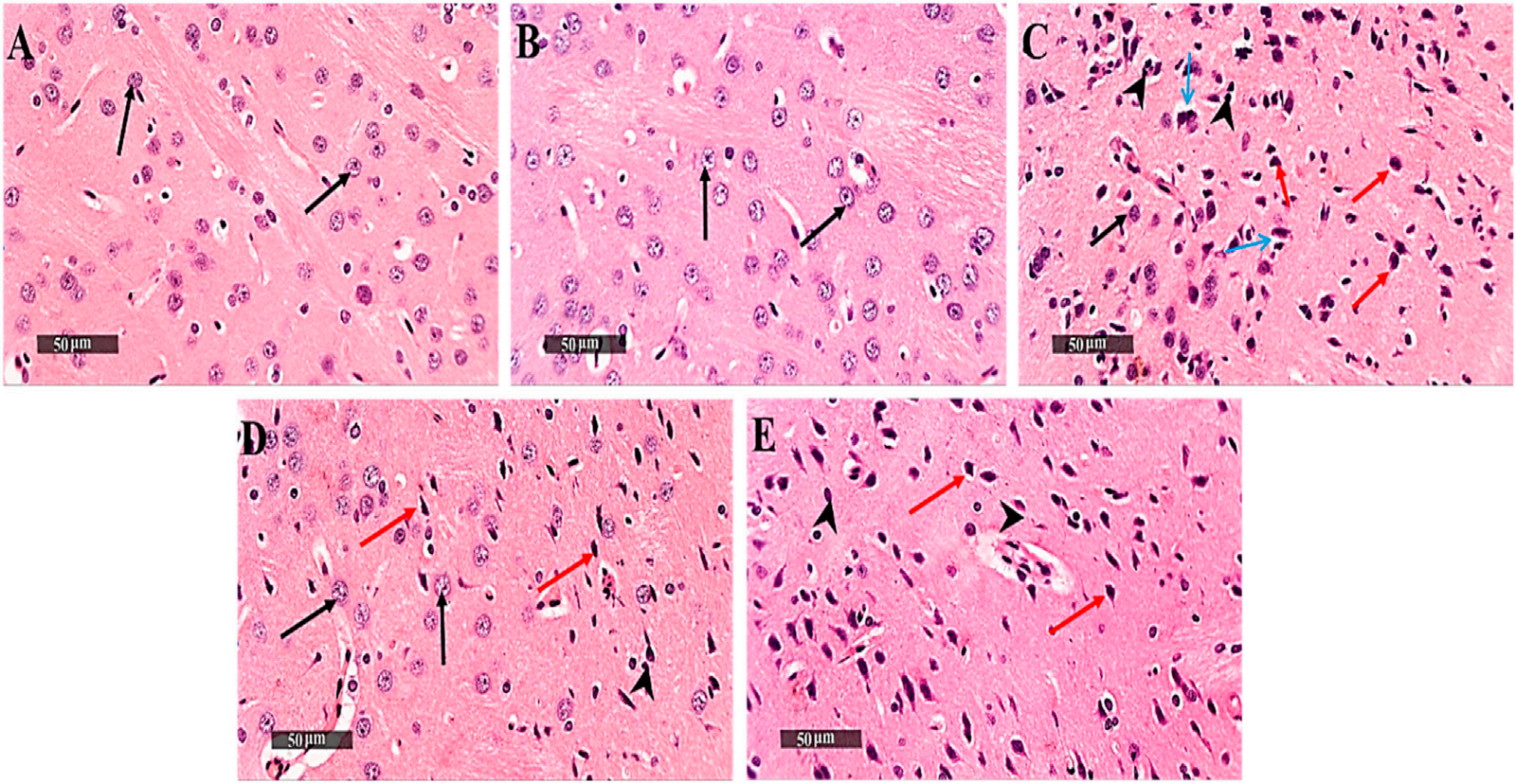
Effect of DAHP on 3-NP induced striatal histopathological alterations. **(A–J)** photomicrographs represent staining of striatum with H&E (Scale bar 200 µm). **(A)** Control group, **(B)** DAHP alone treatment, **(C)** 3-NP group, **(D)** DAHP treated group and **(E)** WM treated group. Well organized apparent intact neurons (black arrow), degenerated neurons (red arrow), perineuronal edema (blue arrow) and severe astrogliosis (arrow head). 3-NP, 3-nitropropionic acid; DAHP, 2,4-diamino-6-hydroxypyrimidine; WM, wortmannin; H&E, hematoxylin and eosin.

Control samples demonstrated normal histological features of striatum region with many well-organized apparent intact neurons with intact subcellular details. On the contrary, 3-NP rats showed severe neuronal loss accompanied with moderate perineuronal edema as well as severe astrogliosis was observed at external lesion border. Interestingly, DAHP treated group showed disappearance of circumscribed lesion records with significant reduction of glial cells infiltrates and appearance of mild records of perineuronal edema. Meanwhile, WM pre-treatment revoked DAHP effects and showed more extensive core lesions than in 3-NP rats (Figure 6).

### 3.7 Effect of 2,4-Diamino-6-Hydroxypyrimidine on 3-Nitropropinic Acid Induced Changes in Striatal Glial Fibrillary Acidic Protein Immunoreactivity

**FIGURE 7:**
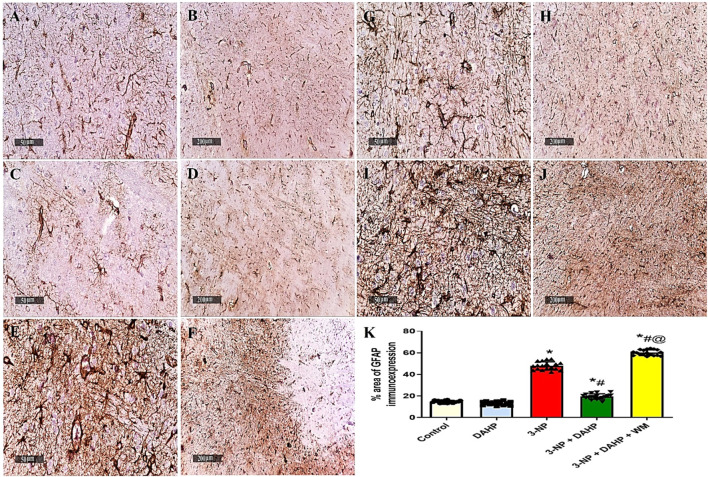
Effect of DAHP on 3-NP induced striatal GFAP immunoreactivity. **(A–J)** photomicrographs represent immunohistochemical staining of GFAP in striatum (Scale bar 50 and 200 µm). **(A–B)** Control group, **(C–D)** DAHP alone treatment, **(E–F)** 3-NP group, **(G–H)** DAHP-treated group and **(I–J)** WM-treated group. **(K)** % area of GFAP immunoexpression. Data are presented as mean ± SD. of three rats per group, using one-way ANOVA followed by Tukey’s post hoc test; F (4.25) = 643; *p* < 0.05. * vs control, # vs 3-NP, @ vs 3-NP + DAHP. 3-NP, 3-nitropropionic acid; DAHP, 2,4-diamino-6-hydroxypyrimidine; WM, wortmannin, GFAP, glial fibrillary acidic protein.

The immunoreactivity of striatal GFAP was assessed by immunostaining as an indicator of the magnitude of astrocyte activation. The 3-NP-treated rats with or without WM showed a significant increase in the immunoexpression of GFAP in striatum along with diffuse astrogliosis. On the other hand, striatal sections from the DAHP-treated group revealed marked reduction in GFAP immunoreactivity to 58.66%, compared with 3-NP-treated rats (Figure 7).

## 4 Discussion

The current study demonstrated the first evidence for the neuroprotective effect of DAHP against 3-NP-induced neurotoxicity in rat model, which was supported by plethora of events; 1) inhibition of GTPCH I activity reduces BH4 biosynthesis resulting in reduction of nitrosative stress and alleviation of mitochondrial dysfunction, 2) activation of MasR/PI3K/Akt/CREB/BDNF/TrKB axis stimulates neurogenesis and suppresses neuroinflammatory status. These positive events were reflected on the behavioral tests showing improvement in motor performance and cognitive impairment, together with reduction in HD symptoms. On the other hand, the use of wortmannin, PI3K inhibitor, reverted the beneficial effects of DAHP.

Striatum is the central core area in the basal ganglia that controls motor coordination, administration of 3-NP produced striatal lesion, which led to motor dysfunction (Jang and Cho, 2016), cognitive impairment (Palfi et al., 1996), and poor retention of memory (Kumar et al., 2007). It was previously reported that 3-NP produces hippocampal lesions in CA1 and CA3 pyramidal neurons; the areas of the brain that is correlated with cognitive performance (Sugino et al., 1999; Kumar and Kumar, 2009). Also, 3-NP increased acetylcholinesterase activity in hippocampus compared with other brain areas suggesting the contribution of hippocampus in cognitive impairment. Furthermore, Borlongan et al. (1997) stated that 3-NP produces cerebral lesions in addition to lesions in other brain areas, including the hippocampus, thalamus, and brain cortex. In the current study, open field, rotarod, Morris water maze, and novel object recognition tests were used to evaluate motor, behavioral, and cognitive abnormalities induced by 3-NP.

Interestingly, DAHP treatment showed significant improvement in locomotor activity, spatial learning, memory retention, and cognitive performance over 3-NP rats indicating the positive effect of DAHP on 3-NP-induced neurotoxicity and degeneration. Moreover, 3-NP induced a significant decrease in the final body weight, which is considered as an indication of 3-NP neurotoxicity (Brouillet et al., 2005). This loss in body weight could be attributed to impairment of energy metabolism, mobilization of energy stores and lipid peroxidation (Pubill et al., 2001). DAHP treatment showed marked improvement in body weight compared with the 3-NP-treated group.

Mitochondrial dysfunction is one of the early pathological hallmarks of HD and one of the basic features of 3-NP model (Carmo et al., 2018). Indeed, 3-NP administration irreversibly inhibits SDH, key enzyme of electron transport chain, leading to inhibition of free fatty acid oxidation and release of massive amounts of reactive oxygen species (ROS) that eventually leads to striatal neurodegeneration (Hariharan et al., 2014; Danduga et al., 2018). Furthermore, 3-NP repressed PGC-1α expression as documented here and earlier (Ahmed et al., 2016). PGC-1α is a transcriptional coactivator that plays an important role in mitochondrial biogenesis and brain energy homeostasis. Accordingly, the suppressed PGC-1α protein expression could be another reason for mitochondrial dysfunction and striatal degeneration observed in 3-NP rats (Chen et al., 2012). Noteworthy, a previous study (St-Pierre et al., 2006) has reported the positive effect of PGC-1α in the expression of several ROS-detoxifying enzymes. Herein, DAHP treatment improved mitochondrial function through increasing SDH level and PGC-1α protein expression. Interestingly, PGC-1α gene possesses cAMP-responsive element (CRE) site for CREB, thus, increased p-CREB observed latter can be the reason for increased PGC-1α expression (Fernandez-Marcos and Auwerx, 2011; Kang et al., 2017).

Regarding oxidative stress, enhancement of Nrf2 protein expression is one of the direct transcriptional targets of CREB (Katoh et al., 2001) that plays a vital role in the defense mechanism against oxidative stress *via* upregulation of antioxidant enzymes, scavenging ROS and enhancement of mitochondrial biogenesis (Satta et al., 2017). In current study, the antioxidant activity of DAHP was witnessed by significant increase in Nrf2 protein expression and SOD content. Of note, a greater protection against H_2_O_2_-induced DNA damage is observed in transgenic animals due to the upregulation of SOD (Reddy et al., 2004). Accordingly, the increase in SOD activity after DAHP treatment elaborates its protective effect against striatal damage caused by oxidative stress.

The 3-NP mimics HD pathogenesis through GABAergic neurons degeneration in the striatum by inducing microglial activation that causes excessive cytotoxic agents production such as nitric oxide, free radicals, and pro-inflammatory cytokines such as tumor necrosis factor-α (TNF-α) and interleukin-1β (IL-1β) (Bonsi et al., 2006; Ahuja et al., 2008). These proinflammatory cytokines have been reported to enhance GTPCH I activity, the rate limiting step for BH4 biosynthesis, that is required as essential cofactor for iNOS activation and NO production (Werner et al., 1993). Subsequently, NO interacts with superoxide to form peroxynitrite (ONOO^_^), a toxic derivative that causes neuronal loss (Saha and Pahan, 2006). Previous studies demonstrated that iNOS has been involved in various neurodegenerative diseases including Alzheimer’s disease, Parkinson’s disease and HD (Dehmer et al., 2004; Lee et al., 2008). Noteworthy, iNOS expression was repressed after DAHP treatment in rat model of MACO due to inhibition of BH4 synthesis (Kidd et al., 2005). Similarly, in our study, DAHP inhibited iNOS expression through inhibition of GTPCH I activity and consequently reduction in BH4 levels, besides reducing TNF-α level following suppression of inflammatory response. Interestingly, in the current study DAHP treatment showed significant inhibition in NF-κB expression through PI3K/Akt pathway activation and suppression of microglial activity. These data is in line with those of Li et al. (2015), who proved that the anti-inflammatory effect of DAHP against cerebral ischemic model is mediated *via* inhibition of NF-κB expression.

Recently, central renin–angiotensin system (RAS) has been involved in the pathogenesis of several neurodegenerative diseases such as PD, AD, and HD (Tian et al., 2012; Rabie et al., 2018; Machado et al., 2020). Of note, Mas receptor (MasR), a RAS component that is expressed in different brain area, has been evoked as counter regulatory arm that opposed the devastating effect of ACE/Ang II/AT1R axis and offered neuroprotective effect through upregulation of PI3K/Akt (Jiang et al., 2013; Rabie et al., 2018). Activated PI3K triggers Akt phosphorylation, this axis entails activation/phosphorylation of CREB (Sakamoto et al., 2011). Phosphorylated CREB plays a substantial role in neurotrophin-mediated neuronal survival *via* transcription of BDNF (Bonni et al., 1999; Sayed et al., 2020) and its receptor TrKB (Song et al., 2015). Worth mentioning, increased BDNF stimulates neurogenesis and triggers TrKB phosphorylation to act as a positive feed-forward loop to re-stimulate MasR and PI3K/Akt axis to promote neuronal survival (Yao et al., 2012; Rabie et al., 2018). Moreover, activated p-TrKB re-activates CREB by phosphorylating it at the S133 site to sustain this cascade (Yoshii and Constantine-Paton, 2010).

Our study revealed that DAHP treatment showed neuroprotective effect against 3-NP *via* increment of MasR protein expression and activation of downstream PI3K/Akt/CREB/BDNF/TrKB cascade. These findings were in line with Li et al. (2015), who stated the neuroprotective effects of DAHP against focal cerebral ischemia was attributed to PI3K/Akt activation. Another evidence to confirm that DAHP neuroprotective effect was mediated through PI3K/Akt pathway was provided by WM, a PI3K inhibitor that abolished histological and biochemical modifications presented by DAHP through blocking the phosphorylation of PI3K and Akt in DAHP-treated rats.

In conclusion, the behavioral, histological, cellular, and neurochemical findings of the current study support for the first time the role of MasR/PI3K/Akt/CREB/BDNF/TrKB pathway activation and iNOS inhibition in the neuroprotective effect of DAHP against neurotoxicity and mitochondrial dysfunction induced by 3-NP, thus, offering a new prospect for the possible role of BH4 inhibitors in HD.

## Data Availability

The original contributions presented in the study are included in the article/Supplementary Material, further inquiries can be directed to the corresponding author.

## Glossary

3-NP: 3-nitropropionic acid
ACE: angiotensin-converting enzyme
Akt: serine–threonine kinase Akt
Ang II: angiotensin II
ARE: antioxidant response element
AT1R: angiotensin type-1 receptor
ATP: adenosine triphosphate
AU: arbitrary unit
BDNF: brain-derived neurotrophic factor
BH4: tetrahydrobiopterin
BSA: bovine serum albumin
CBP: CREB-binding protein
CRE: cAMP-responsive element
CREB: cAMP-responsive element-binding protein
DAB: 3,3′-diaminobenzidine tetrahydrochloride
DAHP: 2,4-diamino-6-hydroxypyrimidine
DMSO: dimethyl sulfoxide
GFAP: glial fibrillary acidic protein
GTPCH 1: GTP cyclohydrolase 1
H&E: hematoxylin and eosin
HD: Huntington’s disease
HTT: huntingtin
iNOS: inducible nitric oxide syntheses
IL**-**1: interleukin-1
MASR: MAS receptor
MCAO: middle cerebral artery occlusion
NF-κB: nuclear factor-κB
NO: nitric oxide
Nrf2: nuclear factor erythroid-2-related factor-2
OFT: open field test
ONOO-: peroxynitrite
PGC-1α: proliferator-activated receptor gamma coactivator 1-alpha
PI3K: phosphoinositide-3-kinase
RAS: renin–angiotensin system
RIPA: radio immunoprecipitation assay
ROS: reactive oxygen species
SDH: succinate dehydrogenase
SOD: superoxide dismutase
TNF-α: tumor necrosis factor-alpha
TrkB: tyrosine kinase B
WM: wortmannin
