# Supplementary figures and images for: Inhibition of Brain GTP Cyclohydrolase I Attenuates 3-Nitropropionic Acid-Induced Striatal Toxicity: Involvement of Mas Receptor/PI3k/Akt/CREB/ BDNF Axis
Source: Front Pharmacol. 2021 Dec 22;12:740966. doi: 10.3389/fphar.2021.740966 (PMC8727546; doi:10.3389/fphar.2021.740966)

**Western Blot Uncropped Figures**

**β-Actin**


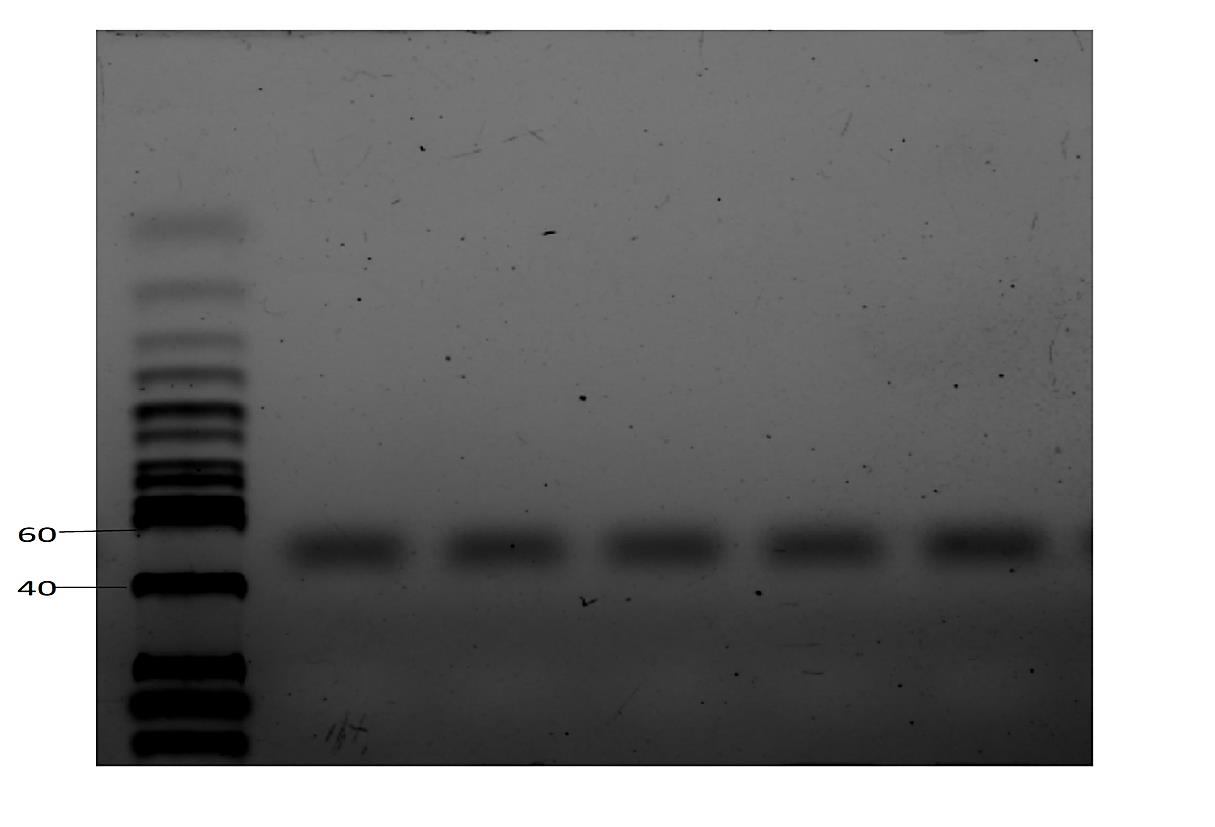


**Mas Receptor**


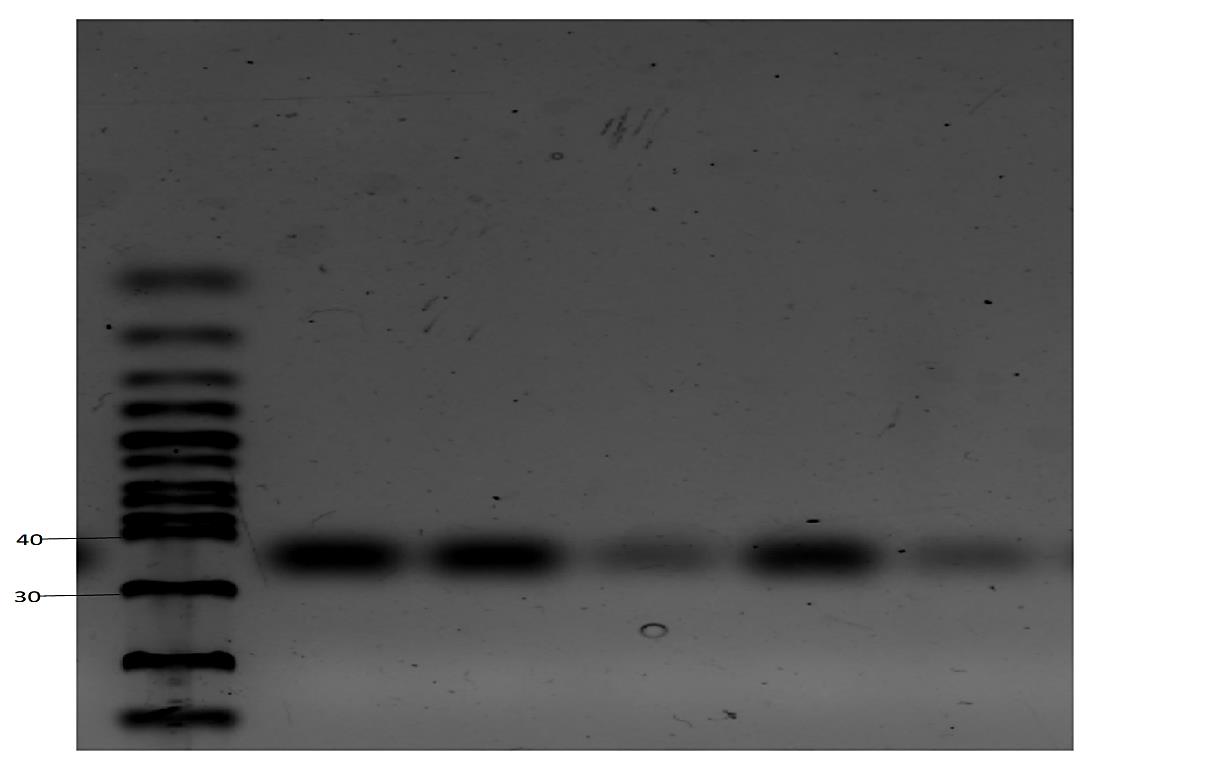


**p85/p55 (pY458/199)-PI3K**


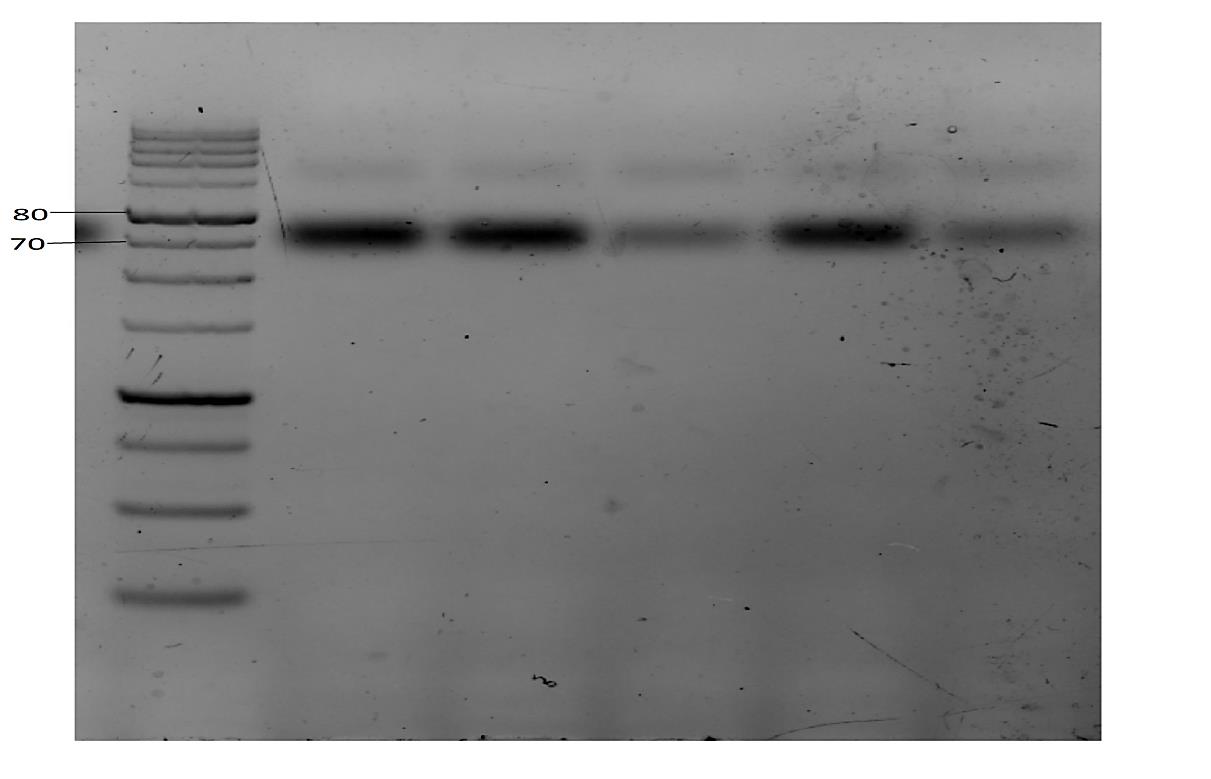


**pS473-Akt**


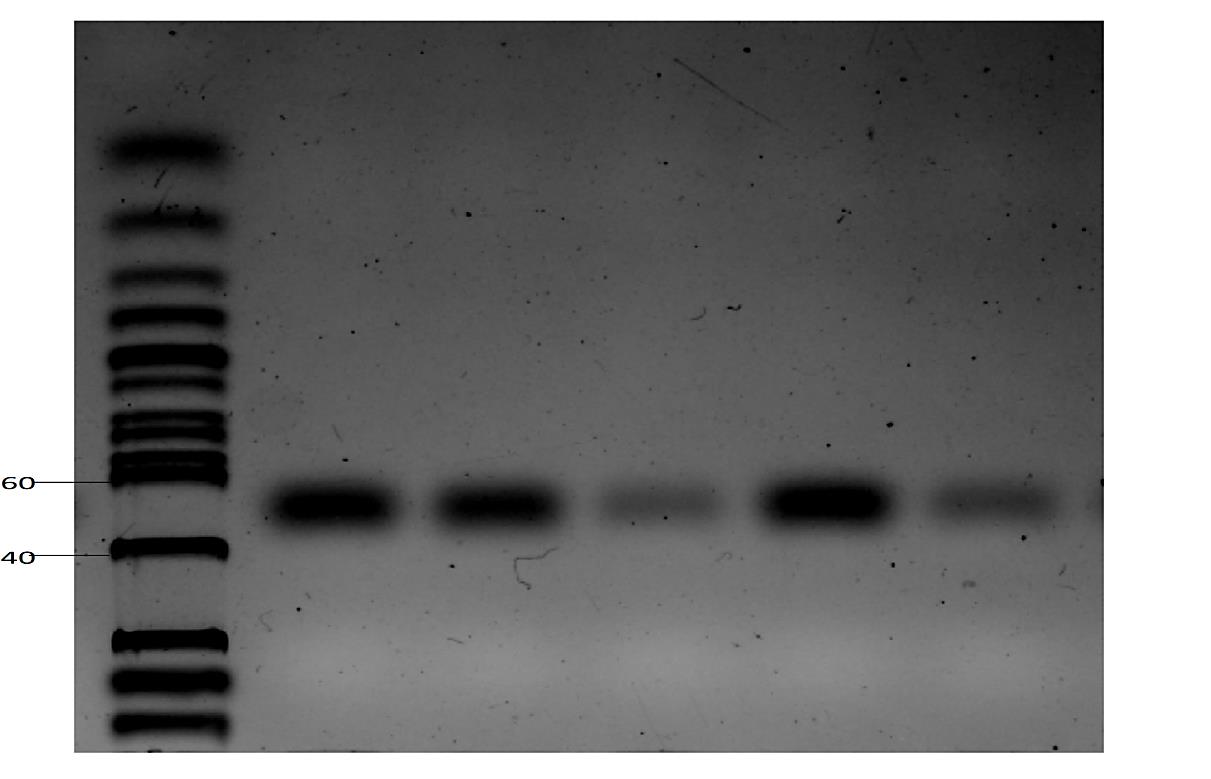


**pS133-CREB**

**
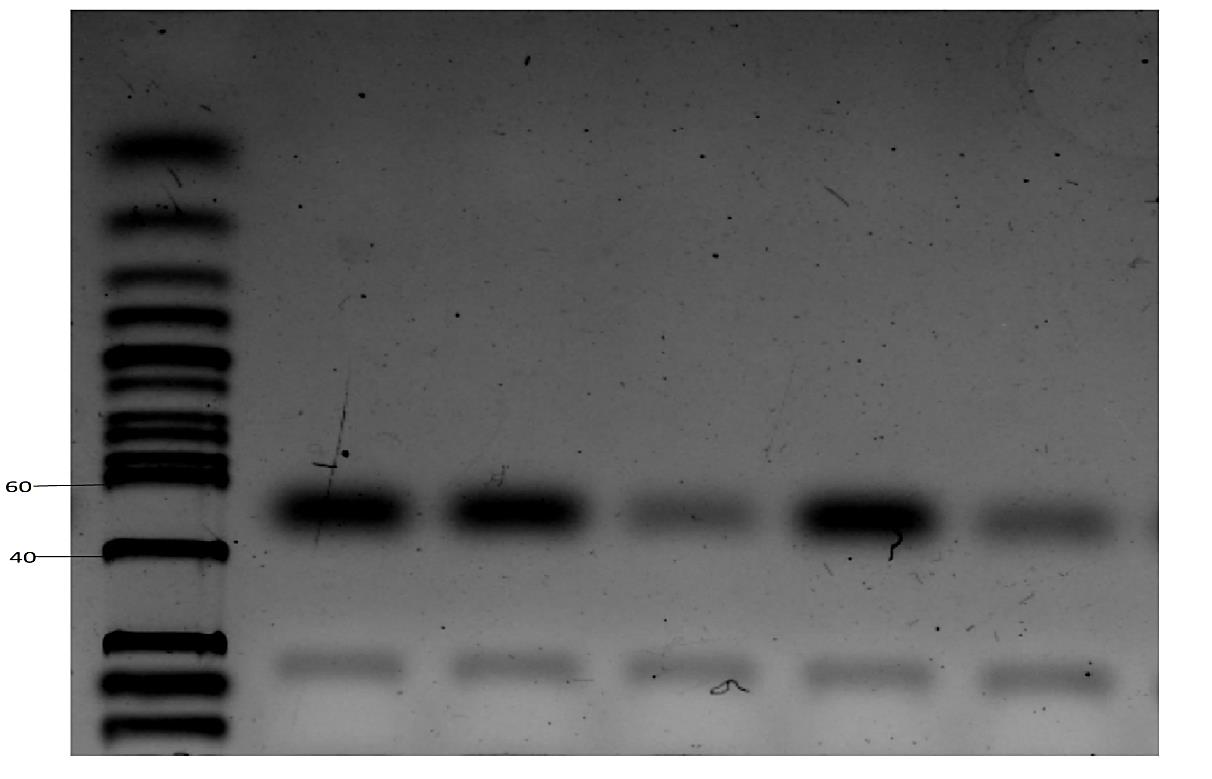
**

**
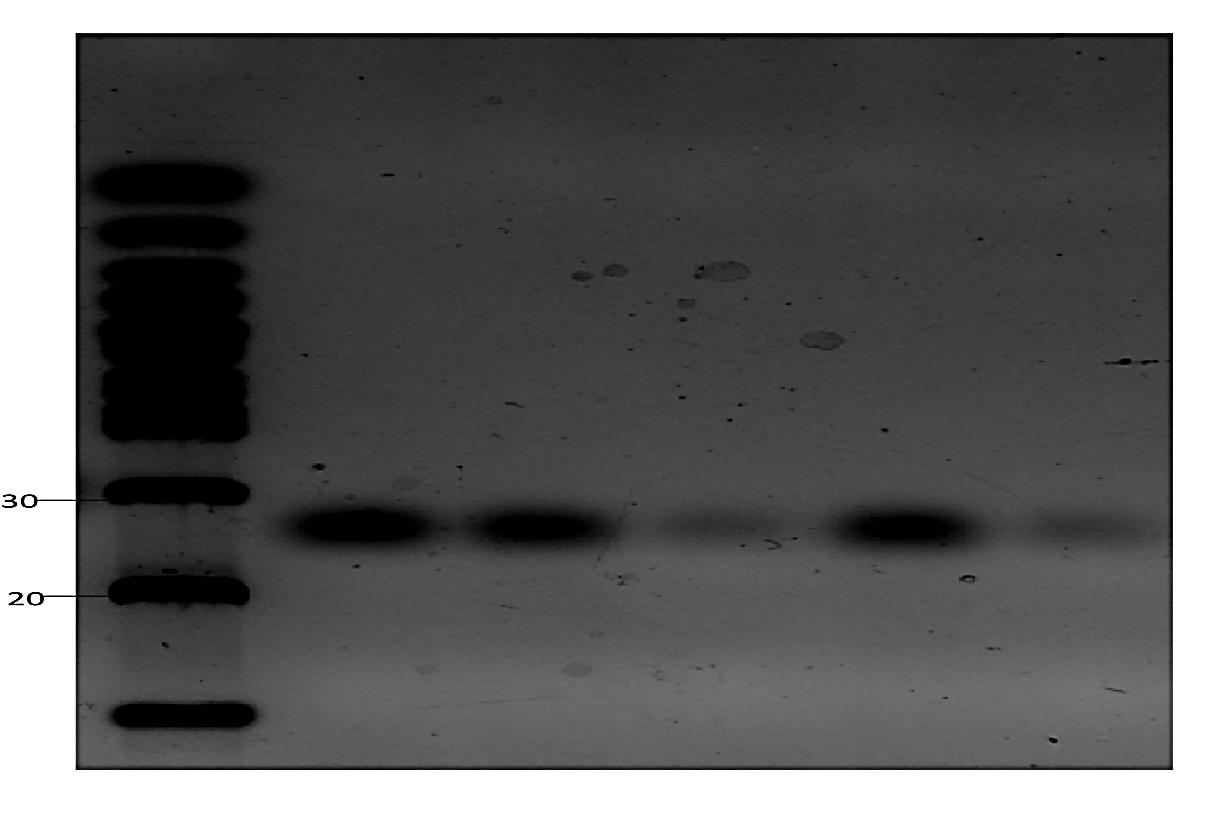
BDNF**

**pY515-TrkB**

**
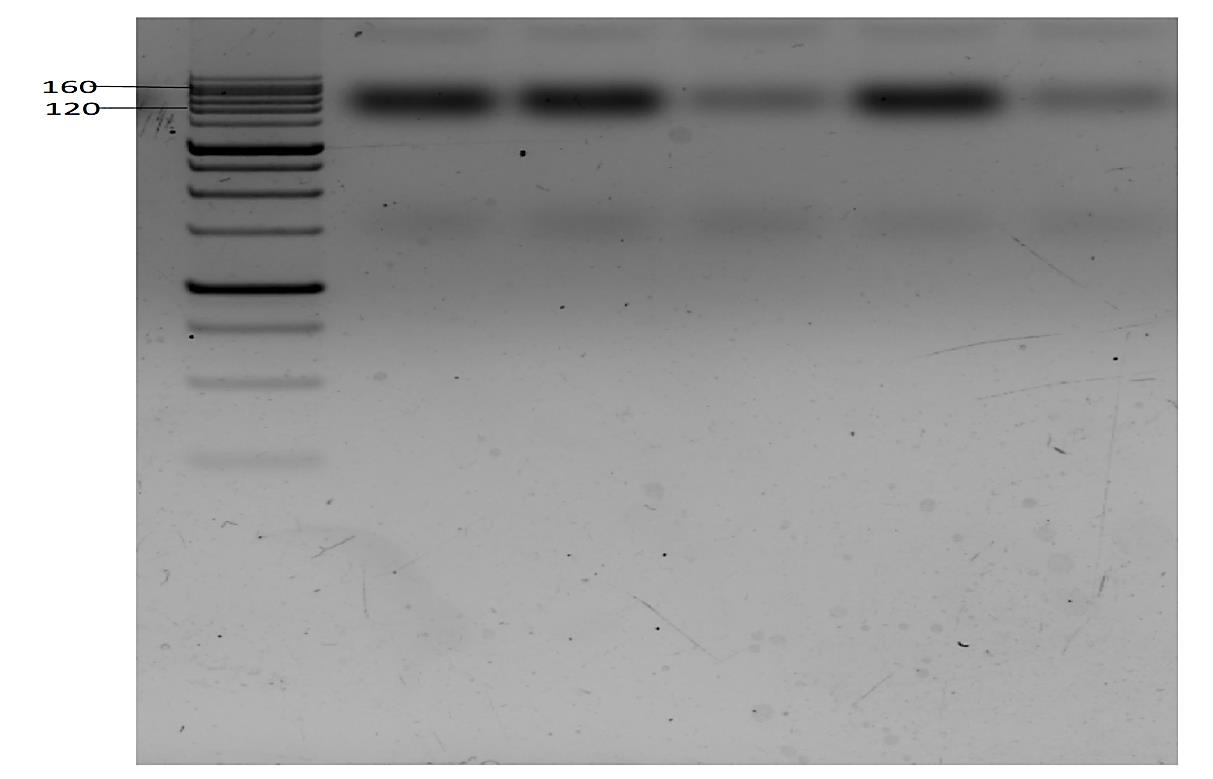
**

**
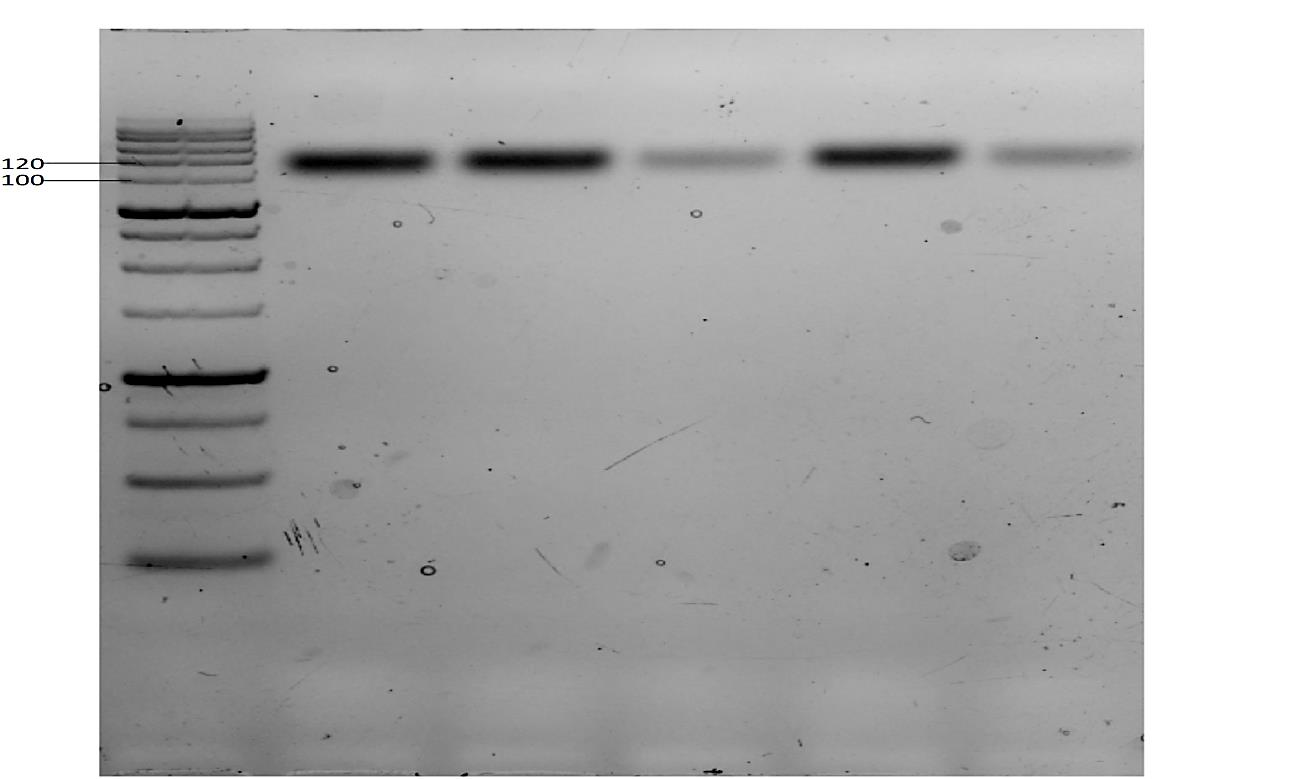
Nrf2**

Supplement: Supplementary file 1 [file Table1.DOCX]
