# Supplementary material for: Inhibition of Brain GTP Cyclohydrolase I Attenuates 3-Nitropropionic Acid-Induced Striatal Toxicity: Involvement of Mas Receptor/PI3k/Akt/CREB/ BDNF Axis
Source: Front Pharmacol. 2021 Dec 22;12:740966. doi: 10.3389/fphar.2021.740966 (PMC8727546; doi:10.3389/fphar.2021.740966)
Supplement: Supplementary file 2 [file Table2.DOCX]

**Highlights:**

- DAHP improved cognitive, memory and motor abnormalities induced by 3-NP.
- DAHP treatment inhibited GTPCH I activity, resulting in decreased BH4 levels and iNOS activation
- DAHP attenuated neuronal loss via activation of MasR/PI3K/Akt/CREB/BDNF /TrKB signaling cascade
- DAHP alleviated mitochondrial dysfunction, as indicated by enhancing both SDH and PGC-1α level and attenuated oxidative stress by increasing SOD activity and Nrf2 expression
- DAHP reduced neuro-inflammatory status by inhibiting NF-κB p65 and TNF-α expression.
